# The Associated Regulatory Mechanisms of Zinc Lactate in Redox Balance and Mitochondrial Function of Intestinal Porcine Epithelial Cells

**DOI:** 10.1155/2020/8815383

**Published:** 2020-12-17

**Authors:** Wenjie Tang, Jing Long, Tiejun Li, Lingyuan Yang, Jianzhong Li, Liuqin He, Shuwei Li, Shengyao Kuang, Yanzhong Feng, Heshu Chen, Fenglan Li, Zhiliang Du, Yulong Yin

**Affiliations:** ^1^Hunan Provincial Key Laboratory of Animal Intestinal Function and Regulation, College of Life Sciences, Hunan Normal University, Changsha 410081, China; ^2^Sichuan Academy of Animal Sciences, Animal Breeding and Genetics key Laboratory of Sichuan Province, Chengdu 610066, China; ^3^College of Animal Science and Technology, Hunan Agricultural University, Changsha, Hunan 410128, China; ^4^CAS Key Laboratory of Agro-ecological Processes in Subtropical Region, Institute of Subtropical Agriculture, Hunan Provincial Key Laboratory of Animal Nutritional Physiology and Metabolic Process, National Engineering Laboratory for Pollution Control and Waste Utilization in Livestock and Poultry Production, Changsha 410125, China; ^5^Heilongjiang Academy of Academy of Agricultural Sciences, Harbin 150086, China; ^6^College of Life Sciences, Northeast Agricultural University, Harbin 150030, China; ^7^Cloud Computing Center, Chinese Academy of Sciences, Dongguan 523808, China

## Abstract

Zinc lactate (ZnLA) is a new organic zinc salt which has antioxidant properties in mammals and can improve intestinal function. This study explored the effects of ZnLA and ZnSO_4_ on cell proliferation, Zn transport, antioxidant capacity, mitochondrial function, and their underlying molecular mechanisms in intestinal porcine epithelial cells (IPEC-J2). The results showed that addition of ZnLA promoted cell proliferation, inhibited cell apoptosis and IL-6 secretion, and upregulated the mRNA expression and concentration of MT-2B, ZNT-1, and CRIP, as well as affected the gene expression and activity of oxidation or antioxidant enzymes (e.g., CuZnSOD, CAT, and Gpx1, GSH-PX, LDH, and MDA), compared to ZnSO_4_ or control. Compared with the control, ZnLA treatment had no significant effect on mitochondrial membrane potential, whereas it markedly increased the mitochondrial basal OCR, nonmitochondrial respiratory capacity, and mitochondrial proton leakage and reduced spare respiratory capacity and mitochondrial reactive oxygen (ROS) production in IPEC-J2 cells. Furthermore, ZnLA treatment increased the protein expression of Nrf2 and phosphorylated AMPK, but reduced Keap1 and p62 protein expression and autophagy-related genes LC3B-1 and Beclin mRNA abundance. Under H_2_O_2_-induced oxidative stress conditions, ZnLA supplementation markedly reduced cell apoptosis and mitochondrial ROS levels in IPEC-J2 cells. Moreover, ZnLA administration increased the protein expression of Nrf2 and decreased the protein expression of caspase-3, Keap1, and p62 in H_2_O_2_-induced IPEC-J2 cells. In addition, when the activity of AMPK was inhibited by Compound C, ZnLA supplementation did not increase the protein expression of nuclear Nrf2, but when Compound C was removed, the activities of AMPK and Nfr2 were both increased by ZnLA treatment. Our results indicated that ZnLA could improve the antioxidant capacity and mitochondrial function in IPEC-J2 cells by activating the AMPK-Nrf2-p62 pathway under normal or oxidative stress conditions. Our novel finding also suggested that ZnLA, as a new feed additive for piglets, has the potential to be an alternative for ZnSO_4_.

## 1. Introduction

Zinc (Zn), one of the most important trace elements in mammals, has been reported to reduce the incidence of diarrhea and improve the structure and function of the intestinal barrier in postweaning piglets [1–4]. Extracellular and intracellular Zn^2+^ in mammalian cells play a key role in physiological or pathological processes, including growth, immunity, and nutrient metabolism [5]. Previous reports have confirmed that Zn deficiency in animals led to a decrease in the number of T cells [6], oxidative stress, intestinal dysfunction, and inflammatory cell infiltration [4, 7, 8]. Traditionally, inorganic Zn (oxides and sulfates) has served as a feed additive to promote growth performance in livestock. To date, Zn additives in the market are in various types, such as zinc oxide, zinc sulfate, and nanozinc, all of which have a benefit in Zn absorption and combating diarrhea [9–13]. However, the excessive use and low absorption efficiency of inorganic Zn in livestock and poultry breeding resulted in the deposition of heavy metals in animal products and the high production of excrement, which inevitably caused concerns in meat safety and environmental pollution [14, 15].

Zinc lactate (ZnLA) is chemically synthesized from feed-grade zinc oxide and *DL*-lactic acid and can easily bind with ligands or metal carriers in enterocytes, which plays a key role in antioxidant function and immune response in animals. Previous studies have reported that the relative bioavailability of ZnLA in animal production is higher than that of inorganic Zn and can improve the growth performance of animals [16]. For example, the addition of ZnLA to animal feed improved the utilization of serum free amino acids and meat quality (e.g., average shell strength and shell thickness) and reduced the shell-breaking rate in chickens [17, 18]. Dietary ZnLA supplementation could also increase the birth weight and weaning survival rate in rabbits, as well as enhance fur elasticity and brightness [19]. Recent reports have indicated that organic Zn in pigs is more helpful in adjusting the adaptive response to piglets' oxidative stress compared with inorganic Zn [20]. However, the effect mechanisms of ZnLA on the antioxidant and anti-inflammatory ability in pigs have not been well-studied.

It is known that nuclear factor erythroid 2-related factor 2 (Nrf2), a principal key transcription factor, has been considered as the main stress regulator that activates the antioxidant system. Upon exposure to various stressors, the release of Nrf2 from Kelch-like ECH-associated protein 1 (Keap1) translocates into the nucleus, resulting in the expression of various cytoprotective genes [21]. Recent studies have reported that Nrf2 could be activated by AMP-activated protein kinase (AMPK) and modulate autophagy-related genes (e.g., p62, Beclin, and LC3B-1/2) to participate in the alleviation of oxidative stress in mammalian cells [22]. Autophagy-related protein p62 can inhibit Nrf2 degradation and promote Nrf2 stability and nuclear translocation by interfering with Keap1-Nrf2 interaction to participate in the cellular antioxidative stress response [23]. However, whether ZnLA could protect against oxidative stress by modulating AMPK-Nrf2 activation and autophagy signals is still poorly understood. Moreover, mitochondria are the main energy source of cells, where they play an important role in cell processes such as apoptosis, reactive oxygen species (ROS) generation, cell cycle, and thermogenesis. Oxidative damage leads to ROS production and mitochondrial dysfunction [24]. A previous study showed that the combination of Zn and selenium improved mitochondrial function and alleviated oxidative stress caused by Alzheimer's disease [24]. Therefore, the purpose of this study was to compare the effects of ZnLA and ZnSO_4_ on cell proliferation and autophagy, Zn transport, antioxidant capacity, and mitochondrial function in intestinal porcine epithelial cells (IPEC-J2) and to reveal the associated regulatory mechanism of ZnLA in H_2_O_2_-induced oxidative stress in IPEC-J2 cells.

## 2. Materials and Methods

### 2.1. Cell Culture

The IPEC-J2 cells derived from the jejunal epithelia of the neonatal piglets were used in all studies to assess the related mechanisms *in vitro*. IPEC-J2 cells were grown in uncoated plastic culture flasks in Dulbecco's Modified Eagle Medium (DMEM), 10% fetal calf serum (FBS; Hyclone, UT, USA), 5 mM L-glutamine, and 1% antibiotics (100 U/mL penicillin and 100 U/mL streptomycin) and cultured at 37°C with 5% CO_2_. The media was changed every two days, and the pH of all cell culture media was maintained at 7.4. The cells covered the bottom of the culture bottle and were trypsinized into a six-well plate and cultured at 37°C with 5% CO_2_. When cells were grown to 70-80% confluence, the cells were cultured in treatment mediums. The cells were then collected to determine the relevant indicators.

### 2.2. Cell Viability Assays

IPEC-J2 cells were seeded in a 96-well plate at a density of 8 × 10^3^ cells/well and grown to 80% confluence. Cells were treated with DMEM containing ZnLA (99%; Sichuan Zoology Feed Co. Ltd.) and ZnSO_4_ with final Zn concentrations of 0, 0.1, 0.5, 1, 2.5, 5, 7.5, 10, 15, and 20 mg/L. After incubation for 6, 12, 24, 36, 48, and 60 h, cell viability was evaluated by cell counting kits (CKK-8) (Dojindo, Kumamoto, Japan) using a microplate reader at 450 nm according to the manufacturer's instructions.

### 2.3. Cell Treatment

At ~70-80% confluence, ZnLA or ZnSO_4_ was added to fresh medium without FBS, which contained the same amount of Zn (7.5 mg/L). In order to eliminate the interference of lactic acid, equal amounts of lactic acid and Zn compared with the ZnLA group were used. To induce oxidative stress, 200 *μ*M H_2_O_2_ (Sigma-Aldrich, MO, USA) was used as previously reported [25]. Compound C (5 *μ*M) (Selleck, Shanghai, China), an AMPK inhibitor, was added to the medium to inhibit AMPK activity.

### 2.4. Intracellular Enzymes and Inflammatory Cytokines

Harvested cells were extracted total proteins; then, cellular malondialdehyde (MDA), superoxide dismutase (SOD), lactic dehydrogenase (LDH), glutathione peroxidase (GSH-PX), interleukin-6 (IL-6), tumor necrosis factor alpha (TNF-*α*), cysteine-rich intestinal protein 1 (CRIP1), cysteine-rich intestinal protein 2 (CRIP2), and metallothionein 1A (MT1A) activities or levels were determined using ELISA kits (Wuhan Huamei Biotechnology Co. LTD) in accordance with the manufacturer's protocols.

### 2.5. Cell Apoptosis Assay

Apoptosis analysis was performed with the Annexin V-FITC/PI (propidium iodide) flow cytometry kit. IPEC-J2 cells were seeded into 6-well plates at a density of 1 × 10^6^ cells/well. After treatment, 5 *μ*L Annexin V-FITC for 15 min and 5 *μ*L PI for 5 min at room according to the manufacturer's instructions [26].

### 2.6. Cell Cycle Assay

Cell cycle progression was examined with a flow cytometer using propidium iodide (PI) staining. Briefly, IPEC-J2 cells were seeded into 6-well culture plates. After treatment, the cells were trypsinized and fixed with cold 70% ethanol at 4°C overnight. The cells were then rehydrated, washed twice with ice-cold PBS, and analyzed by PI staining. PI absorbance was determined by fluorescence-activated cell sorting on a flow cytometer (Beckman Coulter Inc., USA).

### 2.7. Mitochondrial ROS Measurement

Intracellular mitochondrial reactive oxygen (ROS) generation was evaluated using MitoSOX Red reagent (Invitrogen, Shanghai, China). IPEC-J2 cells were seeded into 6-well plates and then cultured in different treatments. Cells were treated with 5 *μ*M MitoSOX Red reagent at 37°C for 10 min in the dark. Then, the fluorescence intensity of 12,000 cells was assayed using a Beckman MoFlo XDP flow cytometer (Beckman Coulter Inc., CA, USA).

### 2.8. Mitochondrial Membrane Potential (MMP) Measurement

Mitochondrial depolarization in the early stages of apoptosis was evaluated using JC-1 reagent (Invitrogen) by double fluorescence staining. The loss of MMP was indicated by a decrease in the red/green mean fluorescence intensity ratio. IPEC-J2 cells were seeded into confocal dishes and then treated under different conditions. JC-1 (10 *μ*g/mL) was added to the medium for 30 min in the dark and then the cells were washed twice with PBS. Cells in the confocal dishes were treated with an antifluorescence quenching agent and observed using a Zeiss LSM880 confocal microscope as previously described [26].

### 2.9. Mitochondrial Respiration Metabolism Assays

Mitochondrial respiration was measured using the XF-24 Extracellular Flux Analyzer and a Cell Mito Stress Test Kit (Agilent Technologies, Inc., CA, USA) in accordance with the manufacturer's instructions. Non-ATP-linked oxygen consumption (proton leak), ATP-linked mitochondrial oxygen consumption (ATP production), and maximal respiration capacity were estimated. Baseline oxygen consumption rate (OCR) minus the maximal respiratory capacity represented the spare respiratory capacity. Residual oxygen consumption after the addition of rotenone and antimycin A was due to nonmitochondrial respiration and was subtracted from all measured values in the analysis. Total cellular protein concentration was determined with a BCA assay kit to normalize mitochondrial respiration rates [27].

### 2.10. Real-Time Quantitative Polymerase Chain Reaction

The expression of mRNA was measured by real-time quantitative PCR. Total RNA was extracted from samples of IPEC-J2 cells using TRIzol reagent (Invitrogen) and reverse transcribed into cDNA using the Prime Script RT reagent kit (TaKaRa Bio, Otsu, Japan). Quantitative PCR was performed using SYBR Premix Ex Taq (TaKaRa Bio, Japan). The reaction was performed at a total volume of 10 *μ*L, with the assay solution containing 5 *μ*L SYBR Green mix (TaKaRa Bio, Japan), 0.2 *μ*L ROX internal reference dye, 3.4 *μ*L deionized H_2_O, 1 *μ*L cDNA template, and 0.2 *μ*L each of the forward and reverse primers. The expression of the housekeeping gene *β*-actin was used to normalize the expression levels. The primers were designed to flank introns using the Primer 5 software. The primer sequences are listed in the supplemental Table 1.

### 2.11. Protein Qualification by the Wes Simple Western System and Western Blot

The process of protein quantification was performed using the Wes Simple Western System (ProteinSimple, San Jose, CA, USA) or the Western Blot technique as previous described [25, 26]. The antibodies used in the study included nuclear factor erythroid 2-related factor 2 (Nrf2) (Abcam, Cambridge, MA, USA), *β*-actin (Abcam), Kelch-like ECH-associated protein 1 (Keap1) (Abcam), AMP-activated protein kinase (AMPK) (Abcam), phosphorylated AMPK (Abcam), lamin B (Abcam), and p62 (Abcam). The mouse *β*-actin antibody was used as a loading control for total protein, while nuclear Nrf2 protein expression was normalized to lamin B. All protein concentrations were determined using a standard BCA protein assay. Results of Wes Simple Western System were obtained using the “gel view” function of the Protein Simple software (ProteinSimple). Western blot data were quantified using the ImageJ software.

### 2.12. Immunofluorescence Assay

IPEC-J2 cells (1 × 10^5^ cells per well) were seeded into confocal dishes and treated with different conditions. Cells were fixed with 4% paraformaldehyde for 20 min and permeabilized with Triton X-100 (0.3%) for 10 min. Then, cells were blocked with bovine serum albumin (1%) for 30 min and were incubated overnight with Nrf2, caspase-3, or Keap1 antibodies diluted at 1 : 100 at 4°C. Cells were washed with cold PBS three times, and then incubated with secondary antibody for 1 h. Nuclear DNA was labeled with 4′,6-diamidino-2-phenylindole (DAPI) for 2 minutes. The fluorescence images were captured using a Zeiss LSM880 confocal microscope and analyzed with the ZEN software.

### 2.13. Statistical Analysis

Statistical analysis was analyzed through one-way ANOVA or *t*-test using the SPSS 19.0 software. All the data were presented as means ± standard error of the mean (SEM). *P* values below 0.05 were considered statistically significant.

## 3. Results

### 3.1. Effects of ZnLA Supplementation on Cell Viability, Cell Cycle, and Apoptosis

**Figure 1:**
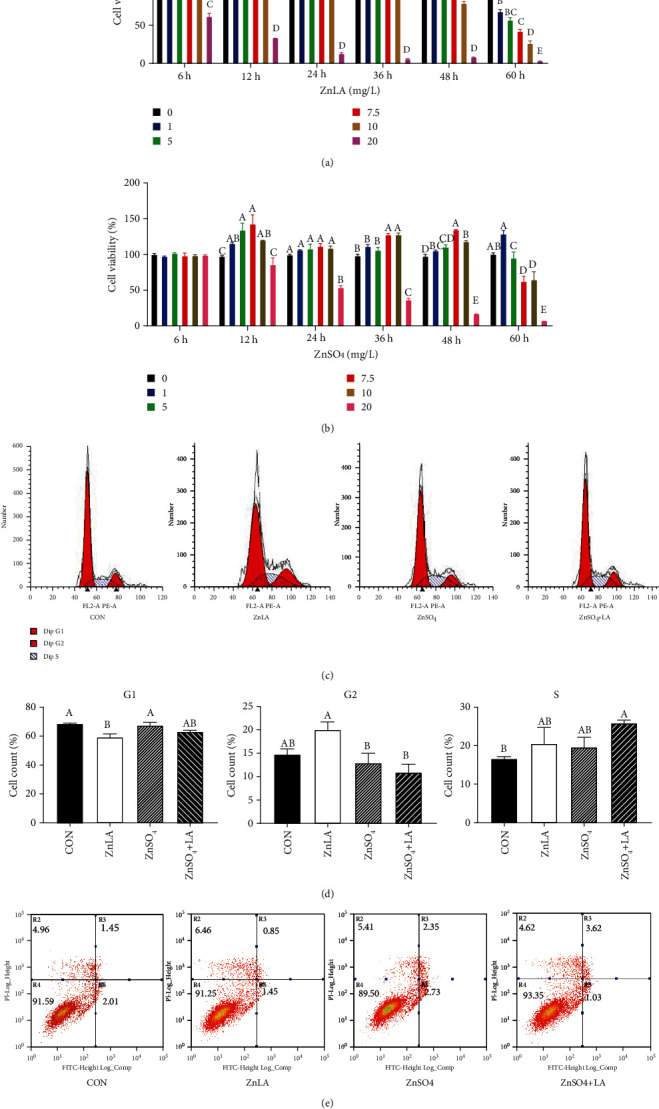
Effects of zinc lactate on cell viability, cell cycle, and apoptosis in IPEC-J2 cells. Values are expressed as means ± SEM (*n* = 4). (a, b) Cell viability under different levels of Zn sources; (c, d) cell cycle in each phase; (e) cell apoptosis ratio. ^a,b,c^Means of bars with different letters were significantly different (*P* < 0.05).

To determine the effects of different Zn sources on cell proliferation in IPEC-J2 cells, we exposed IPEC-J2 cells to increasing concentrations of ZnLA or ZnSO_4_ for 6, 12, 24, 36 48, or 60 h, respectively (Figures 1(a) and 1(b)). We found that exposure to 7.5 mg/L Zn for 12 h significantly increased cell viability compared with other treatments (*P* < 0.05). Thus, the concentrations of 7.5 mg/L Zn from ZnLA or ZnSO_4_ for 12 h were selected as suitable conditions for the subsequent experiments. As shown in Figures 1(c) and 1(d), the G1 phase of the cell cycle was markedly decreased in the ZnLA group compared with the control group (*P* < 0.05). However, ZnLA administration was increased in the S phase (*P* < 0.05) and G2/M phase (*P* < 0.05). In addition, we found that the proportion of early and late apoptotic cells treated with ZnLA was the lowest compared to the other three groups (Figure 1(e)). These results suggested that ZnLA could reduce cell apoptosis and promote cell proliferation.

### 3.2. Effects of ZnLA Supplementation on Mitochondrial ROS, MMP, and Mitochondrial Respiration Metabolism

**Figure 2:**
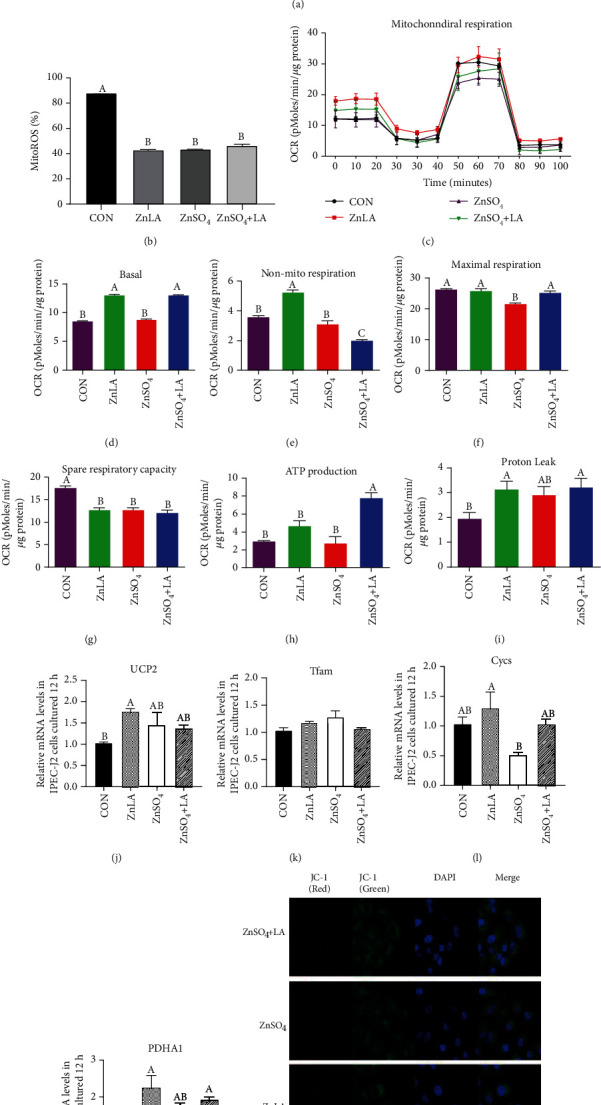
Effect of zinc lactate on mitochondrial ROS, mitochondrial membrane potential, and cellular respiration metabolism in IPEC-J2 cells. Values are expressed as means ± SEM (*n* = 4). (a, b) Mitochondrial ROS level: red line, CON; green line, ZnLA; blue line, ZnSO_4_; yellow line, ZnSO_4_+LA; (c) oxygen consumption rate; (d) basal respiration; (e) non-mito respiratory; (f) maximal respiration; (g) spare respiratory; (h) ATP production; (i) proton leak; (j) the relative expression of UCP2; (k) the relative expression of Tfam; (l) the relative expression of Cycs; (m) the relative expression of PDHA1; (n) mitochondrial membrane potential: red, aggregate; green, monomer. ^a,b,c^Means of bars with different letters were significantly different (*P* < 0.05).

Our results showed that Zn treatment decreased the levels of mitochondrial ROS production (*P* < 0.05) but did not differ between the ZnLA and ZnSO_4_ groups (Figures 2(a) and 2(b)). There was no difference in the ratio of JC-1 red fluorescence to green fluorescence (*P* > 0.05) (Figure 2(n)). Compared with the control group, ZnLA treatment remarkably increased the mitochondrial basal OCR, nonmitochondrial respiratory capacity, and proton leak (*P* < 0.05) (Figures 2(c)–2(i)). Compared with the ZnSO_4_ group, ZnLA administration increased the mitochondrial basal OCR, nonmitochondrial respiratory capacity, and maximal respiration in IPEC-J2 cells (*P* < 0.05). ZnSO_4_+LA administration increased the basal OCR rate, ATP production, and maximal respiration compared with the ZnSO_4_ group (*P* < 0.05). As for mitochondrial-related gene expression, ZnLA supplementation increased the mRNA expression of uncoupling protein 2 (UCP2) and pyruvate dehydrogenase A1 (PDHA1) (Figures 2(j) and 2(m)) compared with the control group, but the mRNA expression of mitochondrial transcription factor A (Tfam) and cytochrome c oxidase (Cycs) was not affected by ZnLA administration (Figures 2(k) and 2(l)). Meanwhile, ZnSO_4_ treatment increased UCP2 mRNA abundance but did not affect the expression of Tfam, Cycs, and PDHA1.

### 3.3. Effects of ZnLA Supplementation on Antioxidant Function, Inflammation, and Zn Transport

**Figure 3:**
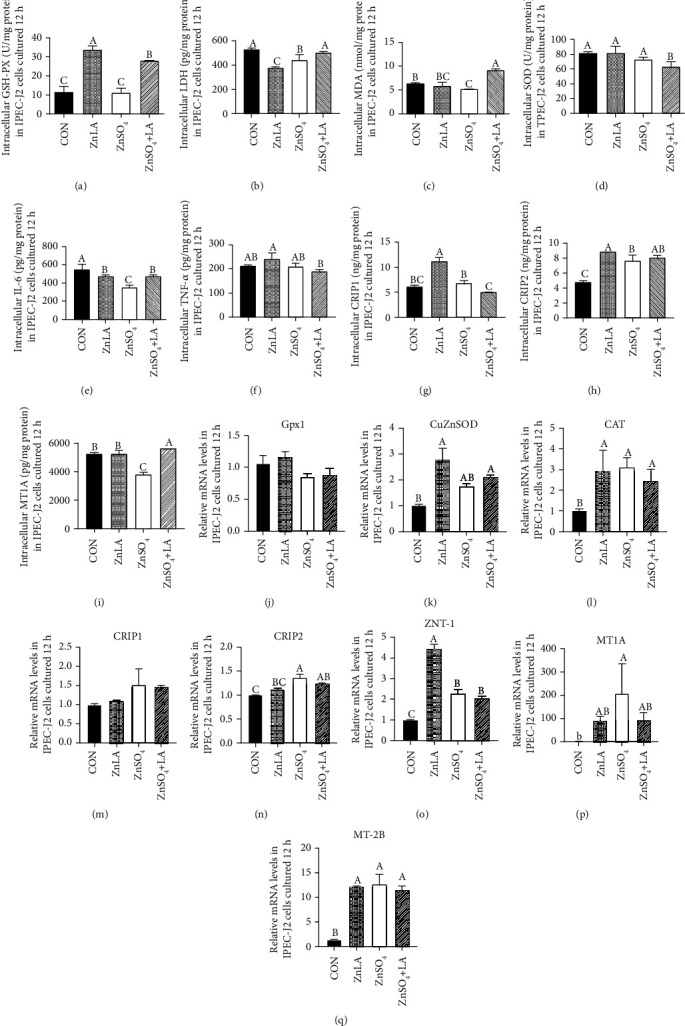
Effect of zinc lactate on Zn transport, inflammatory cytokines, and antioxidant enzymes in IPEC-J2 cells. Values are expressed as means ± SEM (*n* = 4). (a) The activity of GSH-PX; (b) the concentration of LDH; (c) the concentration of MDA; (d) the activity of SOD; (e, f) the concentration of IL-6 and TNF-*α*; (g–i) the concentration of CRIP1, CRIP2, and MT1A; (j–q) the mRNA expression of Gpx1, CuZnSOD, CAT, CRIP1, CRIP2, ZNT-1, MT1A, and MT-2B. ^a,b,c^Means of bars with different letters were significantly different (*P* < 0.05).

For critical validation of the in *vitro* experiment demonstrating the effects of ZnLA on intestinal Zn transport, inflammation, and antioxidant function, we determined the levels or activities of intracellular antioxidant enzymes, inflammatory cytokines, and zinc transporter proteins (Figures 3(a)–3(i)). Compared with the control group, LDH activity was decreased with ZnLA or ZnSO_4_ treatment (*P* < 0.05), and the activity of LDH in the ZnLA treatment was lower than that in ZnSO_4_ treatment. Compared with the control group, ZnLA treatment significantly increased the activity of GSH-PX (*P* < 0.05), while decreasing the MDA concentration (*P* > 0.05). SOD activity in the ZnSO_4_+LA group was the lowest (*P* < 0.05). Compared with the control group, the concentration of intracellular IL-6 in the other three treatments was significantly decreased (*P* < 0.05), but there was no difference in TNF-*α* concentration among these groups. ZnLA supplementation increased the levels of Zn transporter proteins CRIP1 and CRIP2 (*P* < 0.05), but had no effect on MT1A levels compared with that in the CON group. Furthermore, we also determined the mRNA expression levels of Zn transporters and antioxidant-related genes (Figures 3(j)–3(q)). Compared with the control group, ZnLA or ZnSO_4_ supplementation markedly increased the mRNA expression of ZNT-1 and MT-2B in IPEC-J2 cells (*P* < 0.05). The mRNA expression of CRIP2 and MT1A in the ZnSO_4_ group was also increased in comparison with the other three groups (*P* < 0.05), while there was no difference in the expression of CRIP1 among the four groups. The mRNA expression levels of CAT and CuZnSOD in the ZnLA group were higher than those in the other groups, but there was no difference in the expression of Gpx1 among these groups.

### 3.4. Effects of ZnLA Supplementation on the Expression of Nrf2/Keap1, AMPK, and Autophagy-Related Pathways

**Figure 4:**
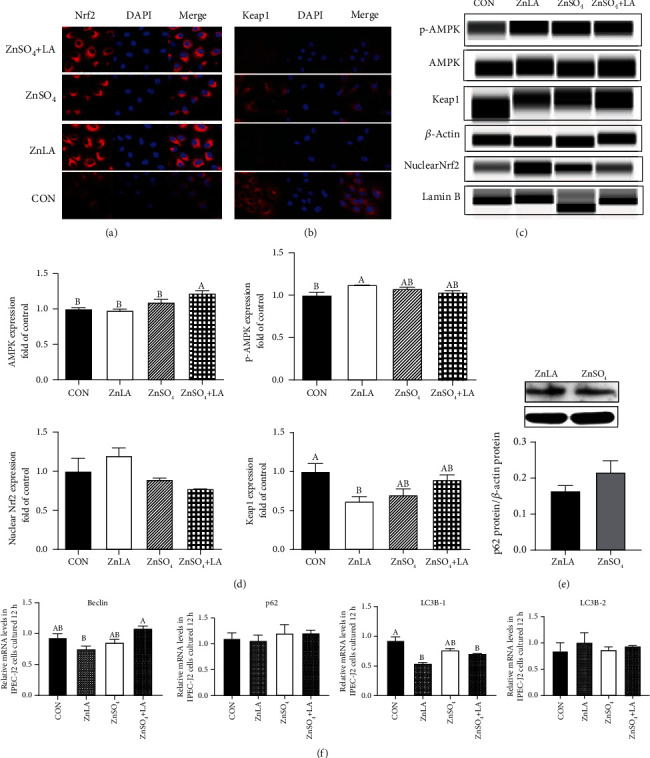
Effects of zinc lactate on the AMPK-Nrf2-p62 signaling pathway in IPEC-J2 cells. Values are expressed as means ± SEM (*n* = 4). (a) Localization of Nrf2 (×63 magnification): red, Nrf2; blue, DAPI; (b) localization of Keap1 (×63 magnification): red, Keap1; blue, DAPI; (c, d) protein expression of AMPK-Nrf2 pathway; (e) p62 protein expression; (f) the mRNA expression of Beclin, p62, LC3B-1, and LC3B-2. ^a,b,c^Means of bars with different letters were significantly different (*P* < 0.05).

To further validate whether ZnLA supplementation could alleviate oxidative stress in IPEC-J2 cells via Nrf2/Keap1, AMPK, and autophagy-related pathways, we determined the expression of the key target molecules using Western blotting and immunofluorescence techniques. We found that Nrf2 protein was mostly located in the cytoplasm of IPEC-J2 cells, but ZnLA administration could increase the amount of Nrf2 transferred to the nucleus (Figures 4(a) and 4(c)). Compared with the control group, the expression of Keap1 was reduced by ZnLA treatment (*P* < 0.05). Furthermore, the protein expression of AMPK in ZnSO_4_+LA group was highest (*P* < 0.05), and ZnLA treatment remarkably increased (*P* < 0.05) the protein expression of phosphorylated AMPK (Figures 4(c) and 4(d)). Compared with the ZnSO_4_ group, the expression of p62 in the ZnLA group was decreased, but there was no significant difference (Figure 4(e)). Our results also showed that ZnLA treatment markedly reduced the mRNA expression of autophagy-related genes LC3B-1 and Beclin (*P* < 0.05), but it had no effect on the mRNA expression of p62 and LC3B-2 (*P* > 0.05) (Figure 4(f)).

### 3.5. Effects of ZnLA Supplementation on Mitochondrial ROS, Apoptosis, and the AMPK-Nrf2-p62-Mediated Pathway under Oxidative Stress Conditions

**Figure 5:**
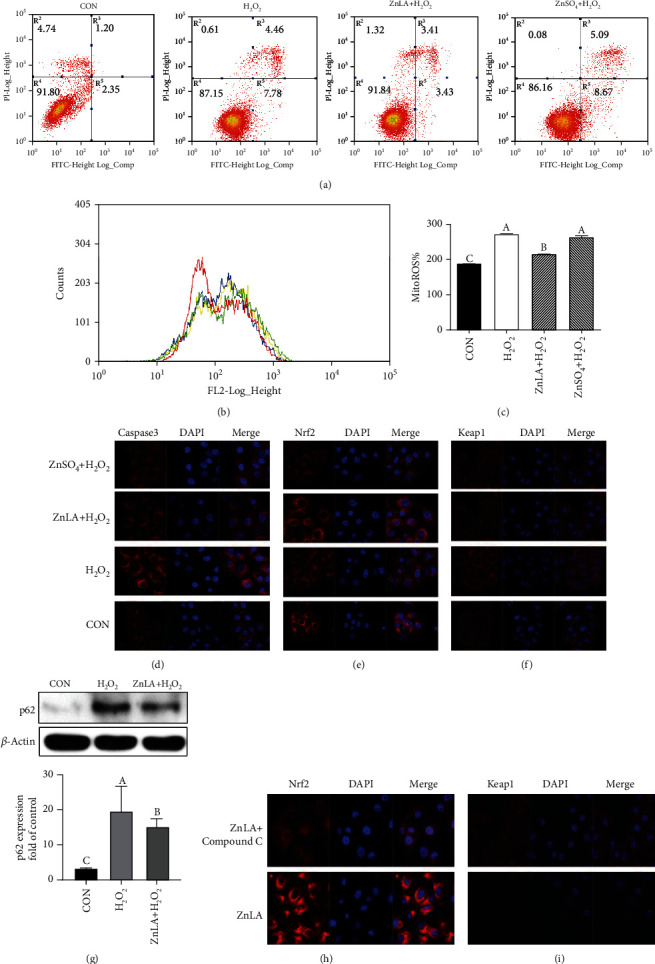
Effects of zinc lactate on cell apoptosis, mitochondrial ROS, and AMPK-Nrf2-p62 signaling pathway in H_2_O_2_-induced IPEC-J2 cells. Values are expressed as means ± SEM (*n* = 4). (a) Cell apoptosis; (b, c) mitochondrial ROS level: red line, CON; green line, H_2_O_2_; blue line, ZnLA+H_2_O_2_; yellow line, ZnSO_4_+H_2_O_2_; (d) caspase-3 expression (×63 magnification): red, caspase-3; blue, DAPI; (e) localization of Nrf2 (×63 magnification): red, Nrf2 or keap1; blue, DAPI; (f) localization of Keap1 (×63 magnification); (g) p62 protein expression; (h, i) localization of Nrf2 and Keap1 (×63 magnification): red, Nrf2 or keap1; blue, DAPI. ^a,b,c^Means of bars with different letters were significantly different (*P* < 0.05).

To further define the effect of ZnLA on the alleviation of oxidative stress in enterocytes, we built an oxidative stress model of H_2_O_2_-induced IPEC-J2 cells. The levels of mitochondrial ROS and apoptosis were determined in the presence or absence of 7.5 mg/L ZnLA. As shown in Figure 5(a), H_2_O_2_ exposure markedly increased cell apoptosis in IPEC-J2 cells, while ZnLA supplementation decreased the proportion of apoptotic cells (the proportion of early apoptotic cells and late apoptotic cells, 6.84%) compared with the H_2_O_2_ treatment groups (12.24%) (*P* < 0.05). The results of the immunofluorescence assay showed that caspase-3 was located in the cytoplasm of IPEC-J2 cells, and compared with the control group, H_2_O_2_ exposure significantly increased caspase-3 protein expression (*P* < 0.05) (Figure 5(d)), while ZnLA or ZnSO_4_ administration decreased the protein expression of caspase-3.

To test whether ZnLA could protect IPEC-J2 cells from oxidative damage by scavenging intracellular ROS, flow cytometry was used to detect mitochondrial ROS. The results showed that compared with the H_2_O_2_ group, ZnLA treatment significantly decreased mitochondrial ROS production in the H_2_O_2_-induced IPEC-J2 cells (*P* < 0.05) (Figures 5(b) and 5(c)). However, ZnSO_4_ treatment had no effect on the levels of mitochondrial ROS in H_2_O_2_-induced IPEC-J2 cells (*P* > 0.05). As shown in Figures 5(e) and 5(f), the protein expression of nuclear Nrf2 in the ZnLA+H_2_O_2_ group was significantly increased while Keap1 protein expression was decreased compared with the H_2_O_2_ group. H_2_O_2_ treatment increased the expression of autophagy-related protein p62, while ZnLA supplementation markedly decreased the expression of p62 in H_2_O_2_-induced IPEC-J2 cells (*P* < 0.05) (Figure 5(g)). To further explore whether AMPK-Nrf2 signaling could be activated by ZnLA supplementation under oxidative stress conditions, we treated cells with an AMPK inhibitor (Compound C) to inhibit AMPK activity [28]. When AMPK activity was inhibited by Compound C, ZnLA supplementation did not promote the nuclear translocation of Nrf2 and did not decrease the protein expression of Nrf2 and Keap1 in the cytoplasm of IPEC-J2 cells (*P* > 0.05) (Figures 5(h) and 5(i)).

## 4. Discussion

Dietary Zn supplementation could promote cell proliferation and protect intestinal barrier function in postweaning piglets against diarrhea [29]. In the present study, we found that the addition of ZnLA was more effective in promoting cell proliferation and suppressing cell apoptosis than ZnSO_4_, at the same concentration. This is consistent with many reports that Zn supplementation plays an important role in improving cell proliferation and differentiation [30, 31]. For example, a recent study has reported that ZnLA supplementation improved the growth performance of young grass carp by maintaining intestinal immune and physical barrier functions [32]. The small intestine, as a major site of Zn absorption, can maintain Zn homeostasis by regulating the expression of Zn transport proteins [33]. A number of proteins involved in Zn absorption and transport have also been identified, including metallothionein (MT), SLC30 (ZNT), SLC39 (ZIP), and CRIP [34]. Previous studies reported that downregulation of ZNT-1 protein could cause the release of LDH and the activation of caspase protein following ischemia-reperfusion [35]. MT participates in the storage, transport, and bioutilization of Zn, so a decreased expression of MT reduces the absorption efficiency of Zn in the body [36]. Moreover, MT2 is rich in reduced thiol groups (SH), which have a free radical scavenging capacity 100 times that of GSH, and can inhibit the release of mitochondrial cytochrome c and activate caspase-3 to reduce cell apoptosis and myocardial injury [37, 38]. These were further confirmed by the present study where it was found that ZnLA administration increased the mRNA expression of ZNT-1, MT1A, and MT-2B and intracellular GSH-PX activity, but decreased LDH activity, cell apoptosis, and caspase-3 protein expression levels in IPEC-J2 cells. Further, CRIP and MT regulate physiological balance by competitive transport of Zn [39]. In the current study, we found that ZnLA supplementation promoted the protein expression of CRIP1/2 in IPEC-J2 cells, suggesting the improvement of Zn transport capacity following ZnLA treatment. The results of cell apoptosis and caspase protein expression also indicated that the antiapoptosis effect of ZnLA was better than that of ZnSO_4_ in IPEC-J2 cells.

Mitochondria, a site for the major source of intracellular ATP, plays a crucial role in scavenging ROS and is tightly linked to apoptosis and proliferation [40, 41]. Our results showed that ZnLA treatment increased the mRNA expression of PDHA1 and UCP2 in IPEC-J2 cells. PDHA1 can regulate mitochondrial ATP production and control the generation of ROS [42]. This is consistent with the results of present study that ZnLA treatment increased mitochondrial ATP production and decreased the production of mitochondrial ROS. UCP2, a protein on the inner membrane of mitochondria, can inhibit mitochondrial membrane transport pore opening, prevent mitochondrial Ca^2+^ overload, and reduce the formation of ROS, thereby inhibiting cell apoptosis [43]. Diano and Horvath reported that UCP2 activation could increase proton leak and then decreased ROS production to defend against oxidative stress [44]. Based on the detection of cell respiration, we also observed that ZnLA administration increased mitochondrial proton leakage, mitochondrial basal OCR, and nonmitochondrial respiratory capacity, suggesting that ZnLA could improve mitochondrial respiratory metabolism and maintain energy equilibrium in IPEC-J2 cells. Our current results showed that ZnLA had no effect on MMP. It is known that decreased MMP promoted mitochondrial membrane permeability transition pore opening, activated the caspase-mediated apoptosis pathway, and led to cell apoptosis [45]. Increased MMP inhibited oxidative phosphorylation, resulting in an imbalance of energy metabolism [46]. Our results indicated that ZnLA administration could maintain the homeostasis of MMP. In addition, our results also showed that ZnSO_4_+LA supplementation increased mitochondrial basal OCR, ATP production, and proton leak. This may be because lactic acid forms pyruvate in the presence of LDH, which then enters the mitochondria to participate in energy metabolism, thereby increasing the production of ATP. These results suggest that ZnLA administration plays important roles in mitochondrial function.

Previous studies reported that dietary Zn deficiency resulted in an increased sensitivity to oxidative stress and increased ROS production in animals [47]. This was evidenced by our findings that treatment with ZnLA improved antioxidant capacity in IPEC-J2 cells by regulating antioxidant-related gene expression and antioxidant enzyme concentrations, as well as reducing mitochondrial ROS levels. GSH-PX, CAT, and SOD are important members of the antioxidant enzyme system [48]. In the present study, ZnLA treatment significantly increased the CAT and CuZnSOD mRNA abundance and the activity of GSH-PX in IPEC-J2 cells, indicating that ZnLA administration may enhance their antioxidant ability by improving the expression and activity of antioxidant-related enzymes. Due to alterations in Zn disposition during the inflammatory response, this makes it even easier to interpret the relationship between Zn metabolism and immune function in animals [49]. Our results showed that ZnLA administration decreased the secretion of proinflammatory cytokines such as IL-6 and TNF-ɑ in IPEC-J2 cells. This is consistent with a previous study as reported that addition of ZnLA could decrease serum IL-6 concentration of grass carp to improve immunity. Recent reports have proved that Zn plays a role in maintaining the integrity of the intestinal mucosa through its function in T cell generation and regulating inflammatory cytokines [50].

It has been reported that antioxidant enzyme activities were partly related to the gene transcription, which were regulated by Nrf2/Keap1 signaling molecules [51]. In the present study, ZnLA administration promoted Nrf2 nuclear translocation and prevented the formation of the Nrf2/Keap1 complex, which resulted in the upregulation of antioxidant gene expression. Bartolini et al. reported that the aggregation of p62 enhanced its interaction with Keap1 and blocked the degradation of Keap1 by autophagosomes, thus activating the translocation of Nrf2 to the nucleus [52]. However, our results showed that under H_2_O_2_ induction conditions, ZnLA supplementation decreased the expression of p62 and Keap1, while increasing the expression of Nrf2. It is possible that the activated Nrf2 signaling pathway inhibited cell autophagy by scavenging ROS, thereby forming an antioxidative stress feedback pathway. Furthermore, there is another evidence showing that activation of AMPK could alleviate oxidative stress via the crosstalk between Nrf2 and AMPK signals [28]. In our current study, Compound C, an AMPK inhibitor, was used to inhibit the activity of AMPK and to investigate the interaction between Nrf2 and AMPK. Our results showed that under normal conditions, the protein expression of phosphorylated AMPK was increased by ZnLA administration, and when the activity of AMPK was inhibited by Compound C, ZnLA treatment still led to a decreased expression of nuclear Nrf2 protein. However, when Compound C was removed, ZnLA administration could significantly increase the expression of Nrf2 and decrease p62 protein expression in H_2_O_2_-induced IPEC-J2 cells. These suggest that ZnLA might activate the AMPK-Nrf2-p62 signaling pathway to alleviate oxidative stress in IPEC-J2 cells. Zimmermann et al. showed that the activation of AMPK could facilitate the nuclear translocation of Nrf2 and improve mitochondrial respiratory metabolism in response to oxidative stress [53]. This is consistent with our current results as showed that ZnLA administration increased the Nrf2 nuclear translocation and AMPK activity as well as cell respiration, thereby promoting the expression of antioxidant-related genes to eliminate excess mitochondrial ROS. These results indicate that exogenous ZnLA may maintain redox balance and mitochondrial function by activating the AMPK-Nrf2-p62 signaling pathway in enterocytes.

## 5. Conclusions

This study provided evidence that the administration of ZnLA has a better effect on promoting mitochondrial ROS against oxidative stress, compared to ZnSO_4_ treatment. Furthermore, ZnLA supplementation enhanced the activities and expression of antioxidant enzymes, decreased proinflammatory cytokine secretion, and modulated mitochondrial function by activating the AMPK-Nrf2-p62 pathway under normal or oxidative stress conditions. The AMPK-Nrf2-p62 pathway activated by ZnLA could further regulate the restoration of redox balance. The *in vitro* efficacy of ZnLA indicated that it may be used in animal trials for the prevention of oxidative stress. Our novel findings also suggested that ZnLA, as a new feed additive for weaned piglets, has the potential to be an alternative for an equivalent amount of inorganic Zn.

## Data Availability

The data used to support the findings of this study are available from the corresponding author upon request.

## Abbreviations

ZnLA: Zinc lactate
Zn: Zinc
Compound C: AMPK inhibitor
MDA: Malondialdehyde
SOD: Superoxide dismutase
LDH: Lactic dehydrogenase
GSH-PX: Glutathione peroxidase
ROS: Reactive oxygen species
MMP: Mitochondrial membrane potential
Nrf2: Nuclear factor erythroid 2-related factor 2
Keap1: Kelch-like ECH-associated protein 1
AMPK: AMP-activated protein kinase
ZNT-1: SLC30A1
MT: Metallothionein
UCP2: Uncoupling protein 2
CRIP: Cysteine-rich intestinal protein
CAT: Catalase
CuZnSOD: Copper-zinc superoxide dismutase
Tfam: Mitochondrial transcription factor A
Gpx1: Glutathione peroxidase 1
PDHA1: Pyruvate dehydrogenase A1
MTF-1: Transcription factor.

## Supplementary Materials

Supplementary Materials Supplemental Table 1 is the primers used for quantitative reverse transcription-PCR.
