# Supplementary material for: The Associated Regulatory Mechanisms of Zinc Lactate in Redox Balance and Mitochondrial Function of Intestinal Porcine Epithelial Cells
Source: Oxid Med Cell Longev. 2020 Dec 17;2020:8815383. doi: 10.1155/2020/8815383 (PMC7762675; doi:10.1155/2020/8815383)
Supplement: Supplementary Materials — Supplemental Table 1 is the primers used for quantitative reverse transcription-PCR. [file 8815383.f1.docx]

**Supplemental Table 1**. Primers used for quantitative reverse transcription-PCR

| AccessionNo. | Gene Primers Product  length(bp) | | |
| --- | --- | --- | --- |
| NM_214301.2 CAT | | | F:CCAGCCAGTGACCAGATGAAG 374  R: ACACCCTTCGCCTTCGAAT |
| XM_003124280.3 β-actin | | | F:CTGCGGCATCCACGAAACT 147  R: AGGGCCGTGATCTCCTTCTG |
| NM_001139470.1 ZNT-1 | | | F:GGCTGAAATTGGACCGGACA 181  R: ACGGAACCCAAAGCATCTCC |
| NM_214201 GPx1 | | | F:TGGGGAGATCCTGAATTG 184  R:GATAAACTTGGGGTCGGT |
| NM_001190422.1 CuZnSOD | | F:TGAAGGGAGAGAAGACAGTGTTAG 181  R:TCTCCAACGTGCCTCTCTTG | |
| NM_214289.1 UCP2 | | F:CTGGGCACCATTCTCACCAT 245  R:AACCGGACCTTTACCACGTC | |
| NM_001130211.1 Tfam | | F:CTCGGGAGCTGCACAAGATT 141  R:CGCAGTCGACTTCCACAAAC | |
| XM_003360244.4  PDHA1 | | F:GCCTAACTCGTCACCCCAAA 155  R:GTGATCCTTGTTTCAGATCCTCG | |
| XM_003355808.4 MT-2B  (MT-2A) | | F:AAAGGAGCCTCGGACAAGTG 118  R:AGCAAACGGGTCAGGTTGTA | |
| NM_001044530.1 Beclin 1  NM_021079895.1 p62  NM_001170827.1 LC3B-1  NM_001190290.1 LC3B-2  NM_001001266.2 MT1A  XM_021081650.1 CRIP1  XM_021081648.1 CRIP2    NM_001129970.1 Cycs | | F:TCCATTACTTGCCACAGCCC 169  R:CCCGATCAGAGTGAAGCTGT  F:AATCATGTCCATCCCCAACCTCC 107  R:CCTCACACTCCAGCAGCGTCA  F:CCTCAGACCGGCCTTTCAA 124  R:TGCTTCTCACCCTTGTAGCG  F: GCCGAACCTTCGAACAGAGA 190  R:AGCTGCAAGCGCCTTCTAAT  F:GCCCTGCCCCAGATGTAAATA 86  R:AAGGGATGTAGCATGAAGTCAG  F: TACTTTGCTGAGCGGGTGAC 195  R: GAAGGTGTGACTCTCGGCTC  F: CGCTGCAACAAGAGGGTCTA 161  R: ATTCCGTAGCAGGGCTTGTG  F:TAGCCTGTTAAGGCATGAGCC 343  R:CACACACATCCGGGAGTTCT | |
